# Supplementary material for: TbD1 deletion as a driver of the evolutionary success of modern epidemic Mycobacterium tuberculosis lineages
Source: Nat Commun. 2020 Feb 4;11:684. doi: 10.1038/s41467-020-14508-5 (PMC7000671; doi:10.1038/s41467-020-14508-5)
Supplement: Supplementary file 1 — Supplementary Information [file 41467_2020_14508_MOESM1_ESM.pdf]

TbD1 deletion as a driver of the evolutionary success of modern epidemic *Mycobacterium tuberculosis* lineages

Daria Bottai, Wafa Frigui, Fadel Sayes, Mariagrazia Di Luca, Dalila Spadoni, Alexandre Pawlik, Marina Zoppo, Mickael Orgeur, Varun Khanna, David Hardy, Sophie Mangenot, Valerie Barbe, Claudine Medigue, Laurence Ma, Christiane Bouchier, Arianna Tavanti, Gerald Larrouy-Maumus, and Roland Brosch

Supplementary Figure 1. Virulence of *M. tuberculosis* “Indian” strains in the guinea-pig infection model. Photographs of lungs and spleens recovered form *M. tuberculosis* 79112-, Tb36- and 79499-infected guinea pigs, 56 days after infection. Note that 79112 and Tb36 are TbD1-intact “ancestral” *M. tuberculosis* strains, whereas strain 79499 corresponds to a TbD1-deleted “modern” *M. tuberculosis* strain.

For each strain, organs from a representative infected-animal are shown.

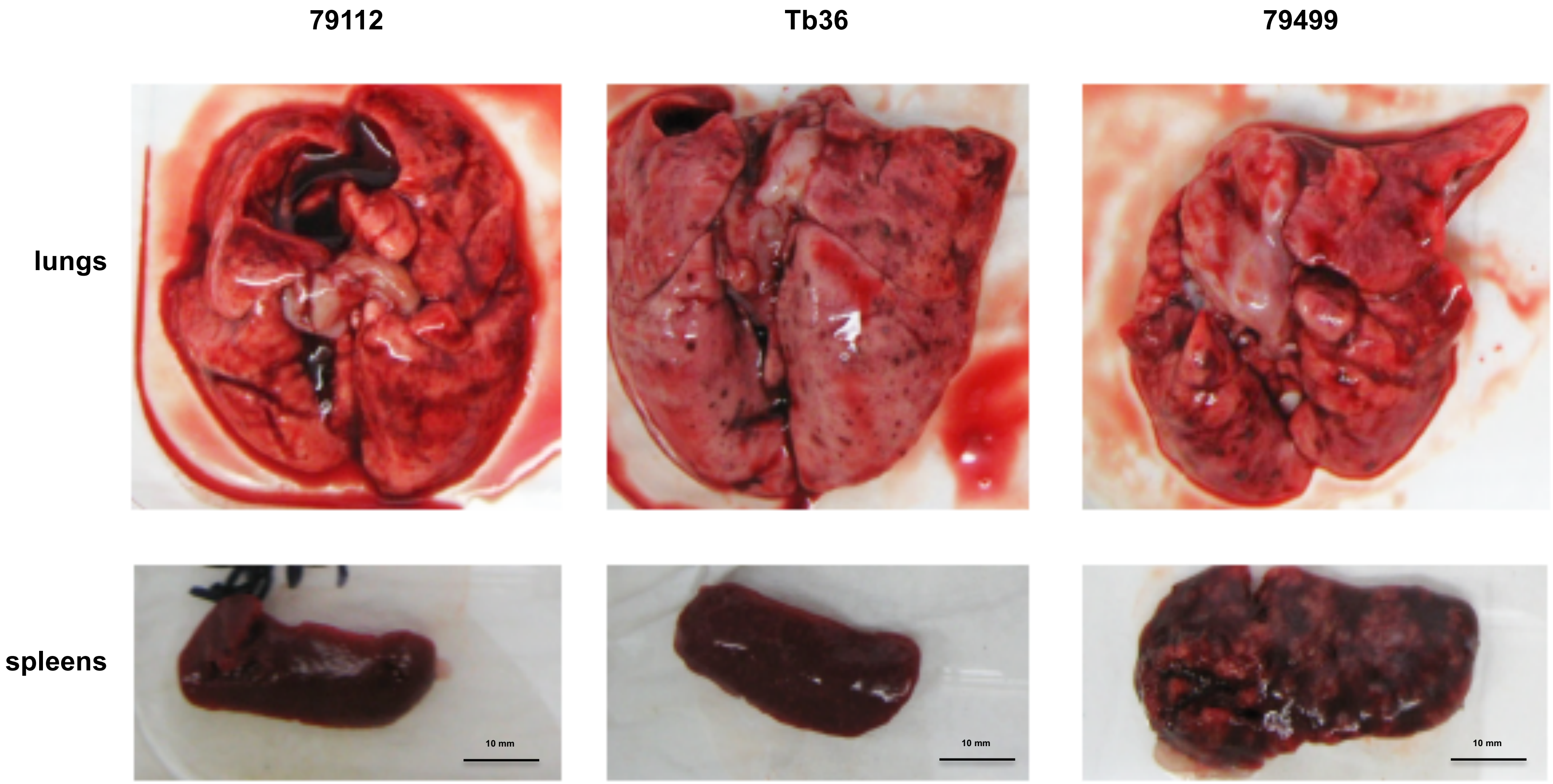

Supplementary Figure 2. Intracellular growth profiles of selected Indian strains in ex vivo models.

Comparative analysis of growth of wild-type TbD1-intact and TbD1-deleted *M. tuberculosis* strains in bone marrow derived macrophages (BMDM) (a and b), BAL-derived guinea-pig macrophages (c and d), human THP-1 (e and f) and A549 (g and h) cell lines.

In all ex vivo models, the number of intracellular bacteria (CFU) was determined immediately after phagocytosis and at different time points (as indicated in the corresponding figure panels a, c, e, g). CFU ratio values (CFU at different time points / CFU at day 0) are also reported in figure (panels b, d, f, h). The figure shows single data points, mean, and standard deviations of values (CFU and CFU ratio) obtained in a representative experiment performed in triplicate (panel a-c) or in six independent tests (panels e-h). The statistical significance of differences in CFU and CFU ratio values between Indian strains and the H37Rv control was determined by one-way Anova with Bonferroni post hoc test (*: P< 0.05; **: P< 0.01).

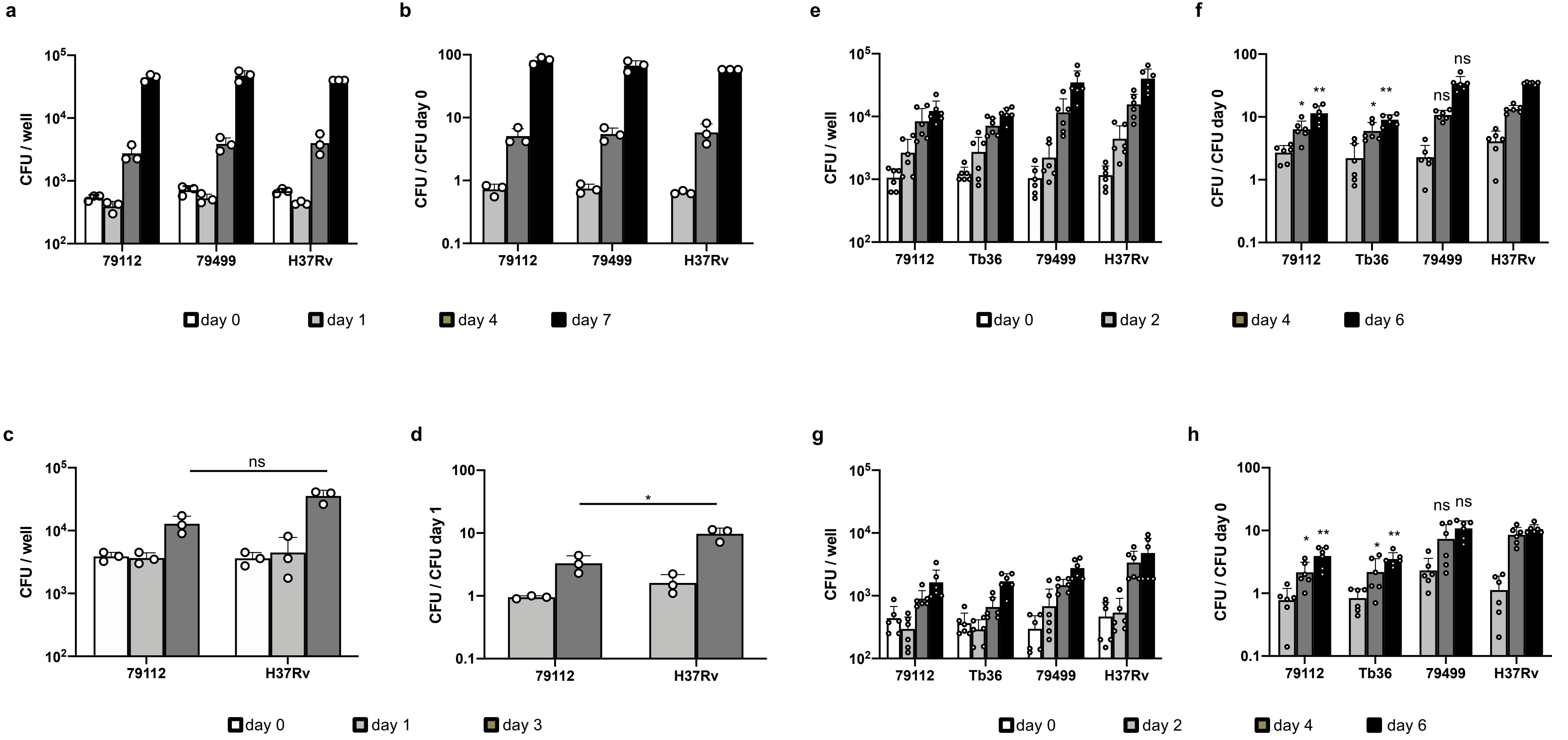

Supplementary Figure 3. Construction of the 2G12 cosmid used for integration of the intact TbD1 locus into 79112ΔTbD1 and H37Rv strains. The *M. tuberculosis* Tb36 genomic fragment encompassing the TbD1 locus and flanking regions was cloned into the pYUB412 intergative cosmid. The obtained recombinant cosmid 2G12 was then used to transform *M. tuberculosis* 79112ΔTbD1 and H37Rv strains.

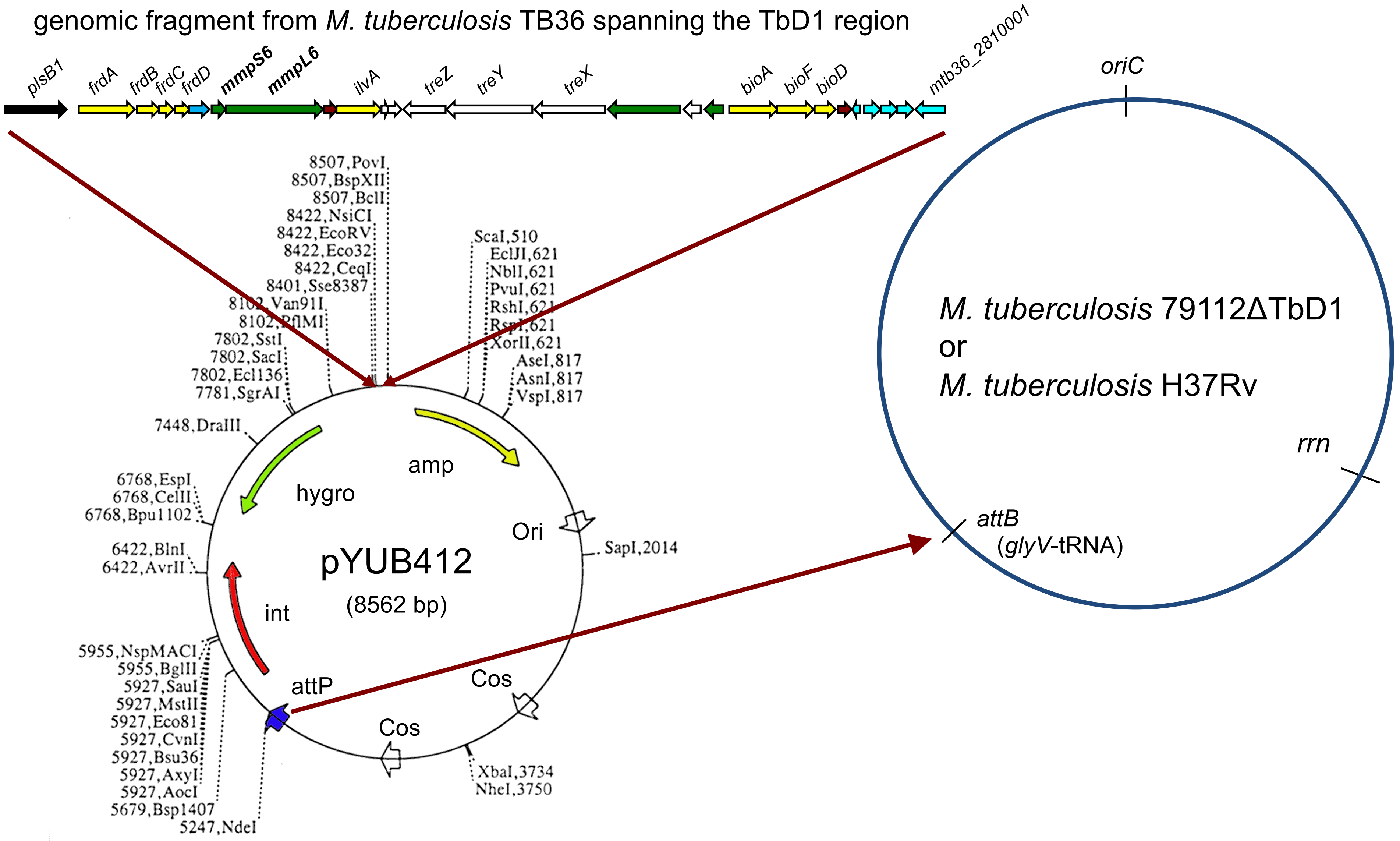

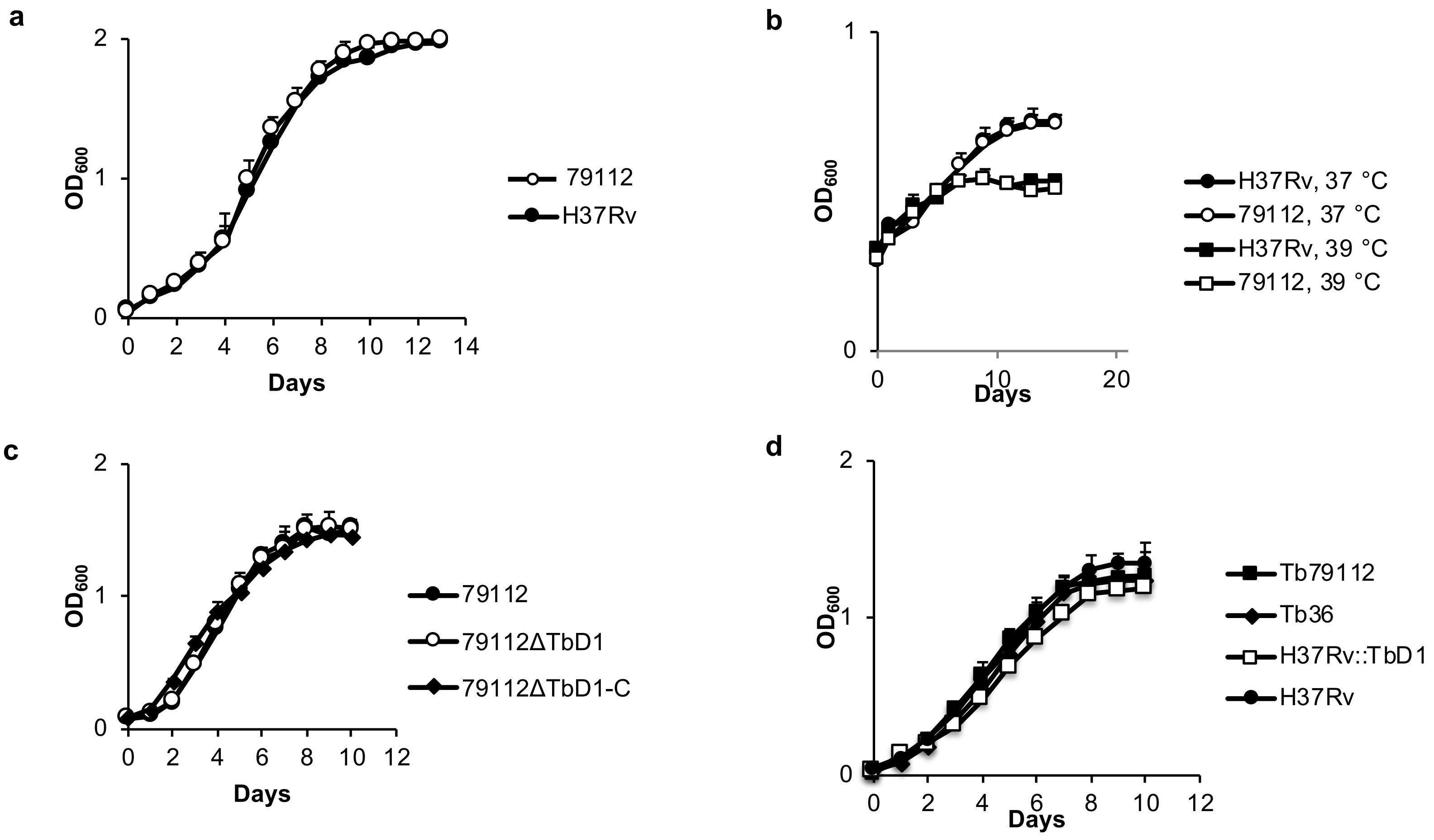

Supplementary Figure 4. In vitro growth characteristics of *M. tuberculosis* 79112- and H37Rv-derivative strains. *M. tuberculosis* 79112 and H37Rv strains, and their corresponding TbD1-deleted and/or TbD1-complemented strains in logarithmic growth-phase were diluted in different fresh media to an initial O.D600 of 0.02 (a), 0.05 (b), 0.08 (c) and 0.02 (d) in tubes with an air volume: liquid volume ratio of 5:1. In all conditions, the bacterial growth was monitored daily by measurement of the O.D600 of cultures. a. Growth of 79112 and H37Rv strains in Middlebrook 7H9 medium, at 37°C, in shaking conditions. b. Growth of 79112 and H37Rv strains in Middlebrook 7H9 medium, at 37°C or 39°C, in standing conditions. c. Growth of 79122ΔTbD1 mutant and its corresponding 79112 and 79112ΔTbD1-C control strains, in Dubos medium without glycerol, at 37°C, in aerobic conditions. d. Growth of H37Rv-TbD1 complemented strain in Dubos medium without glycerol, at 37°C, in aerobic conditions. Each panel reports the mean and the standard deviations of OD_{600} values measured in 3 (panel a, b and d) or 4 (panel c) independent growth assays.

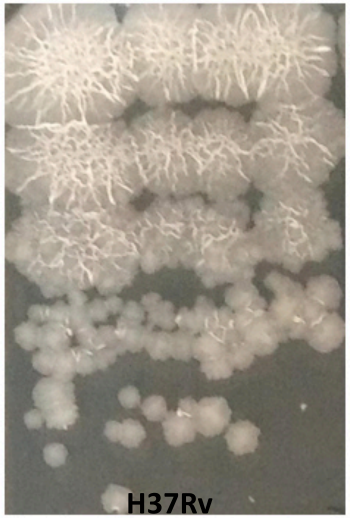

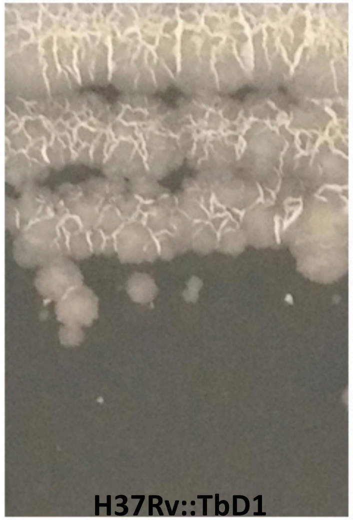

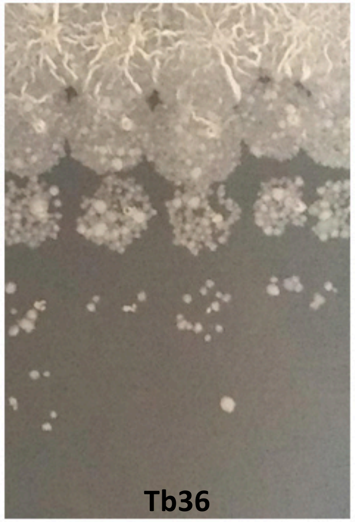

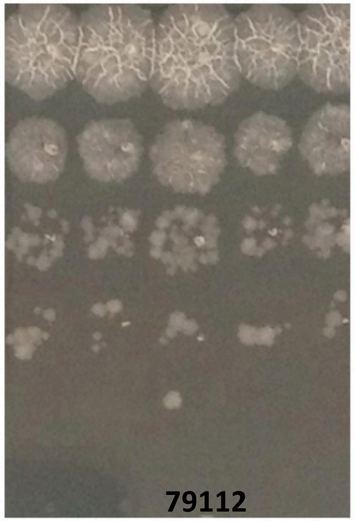

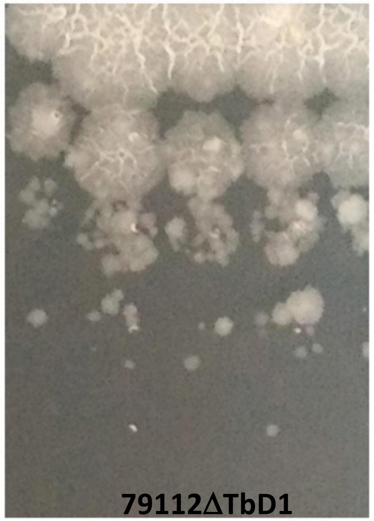

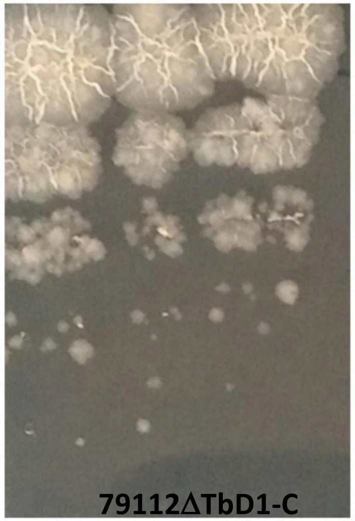

Supplementary Figure 5. Colonies form *M. tuberculosis* TbD1-intact and TbD1-strains on 7H11 Middlebrook agar, 4 weeks post inoculation

Supplementary Figure 6. Virulence of 79112 TbD1-deleted derivative and H37Rv TbD1-complemented *M. tuberculosis* strains in guinea pigs. **a.** Photographs of lungs and spleens recovered from *M. tuberculosis* 79112-, 79112ΔTbD1-, 79112ΔTbD1-C- and H37Rv-infected guinea pigs, 56 days after infection are shown.

b. Photographs of lungs and spleen recovered from Tb36-, H37Rv::TbD1- and H37Rv-infected guinea pigs, 56 days after infection.

For each strain, organs from two representative infected-animals are shown.

a

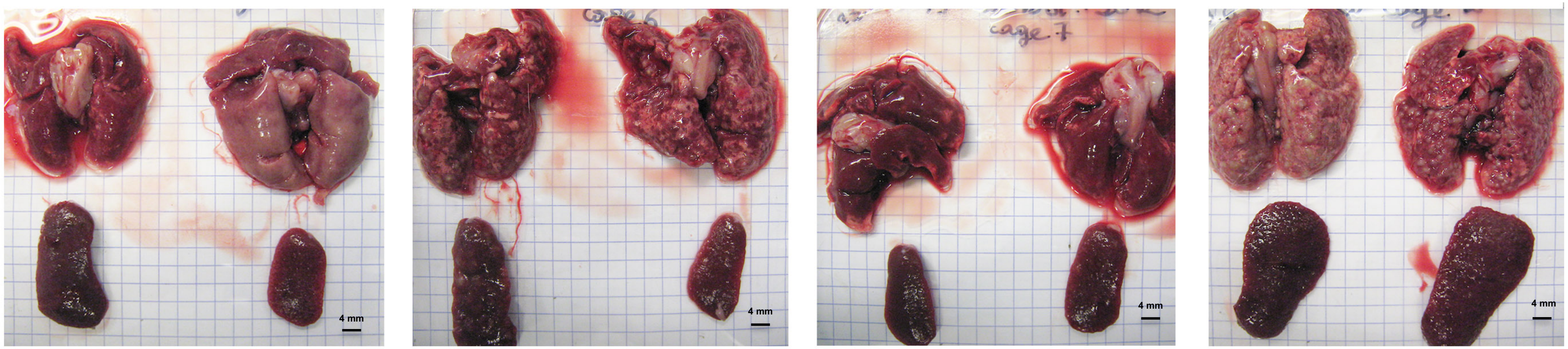

79112 **79112ΔTbD1** **79112ΔTbD1-C** **H37Rv**

b

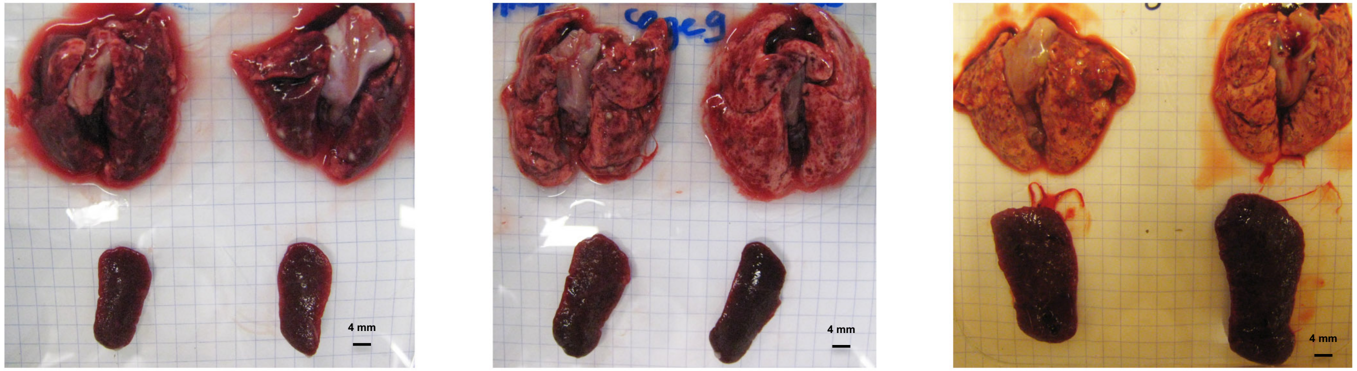

Tb36 **H37Rv::TbD1** **H37Rv**

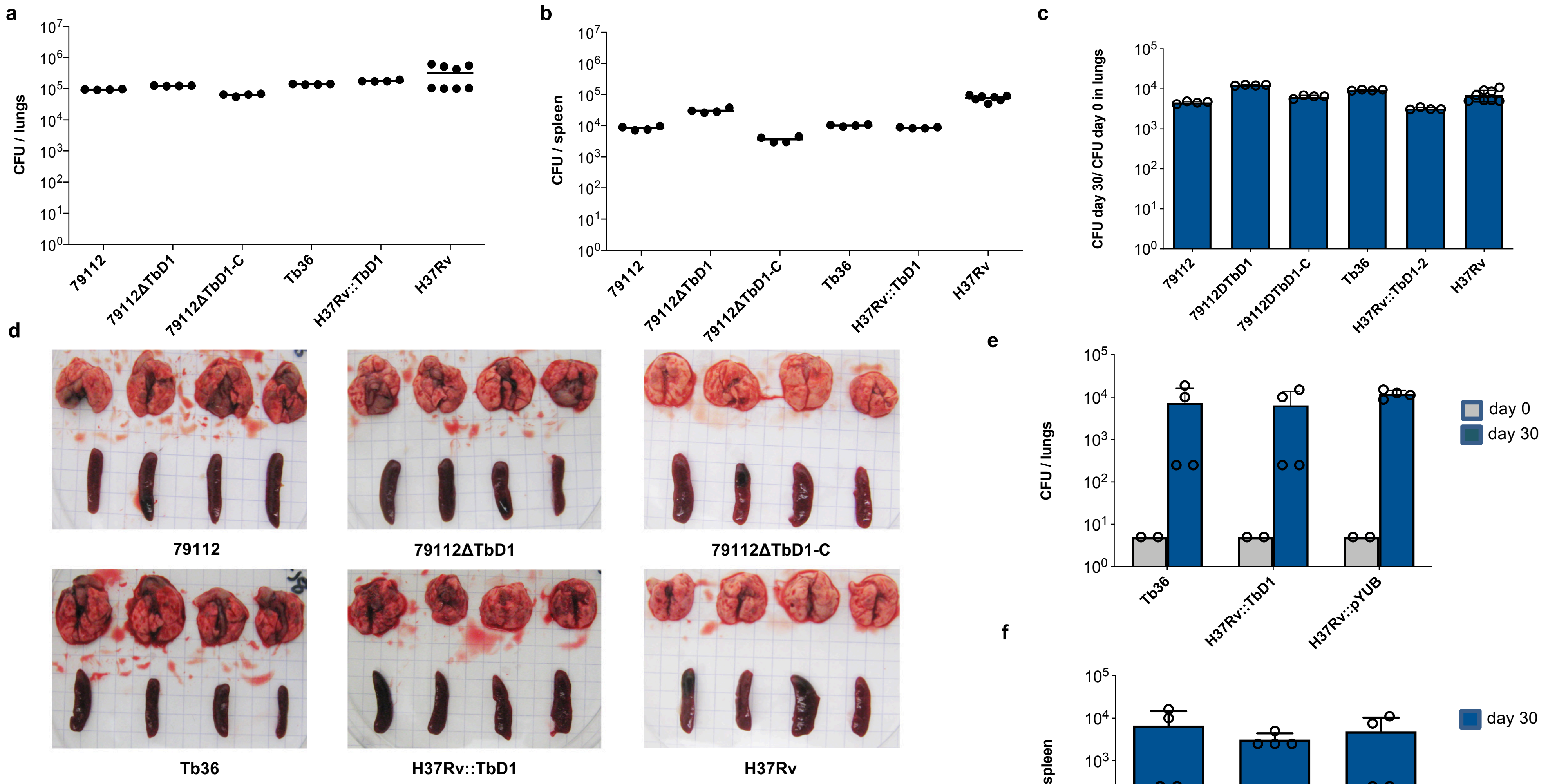

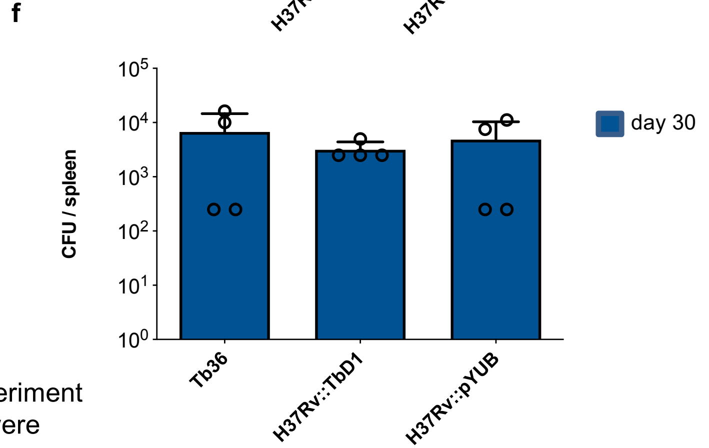

Supplementary Figure 7. Virulence profiles of TbD1-mutant and TbD1-complemented *M. tuberculosis* strains in the standard C57BL/6 mouse infection model. **a-d.** C57BL/6 mice were aerosol infected with different *M. tuberculosis* variants, to deliver an infectious dose at day 0 of 10-50 CFU/mouse. Thirty days after infection, the bacterial load in lungs **(a)** and spleen **(b)** of infected mice was determined.

c. CFU ratio (CFU at day 30/ CFU at day 0) obtained in lungs of mice infected with TbD1-mutant or TbD1-complemented *M. tuberculosis* variants. Panels a, b and c depict data obtained in a representative experiment performed with 4 mice per group (8 for the H37Rv strain). **d.** Photographs of lungs and spleens of mice that were Infected with TbD1-intact or TbD1-deleted *M. tuberculosis* strains, four weeks after infection.

e and f. Evaluation of virulence of *M. tuberculosis* TbD1-complemented strain in comparison with *M. tuberculosis* wild-type strain, complemented with the empty vector pYUB412. Four C57BL/6 mice were aerosol infected with different *M. tuberculosis* strains (estimated infectious dose at day 0 of 5-10 CFU/mouse).
The bacterial load in lungs (**e**) and spleens **(f)** of infected mice was determined four weeks after infection.

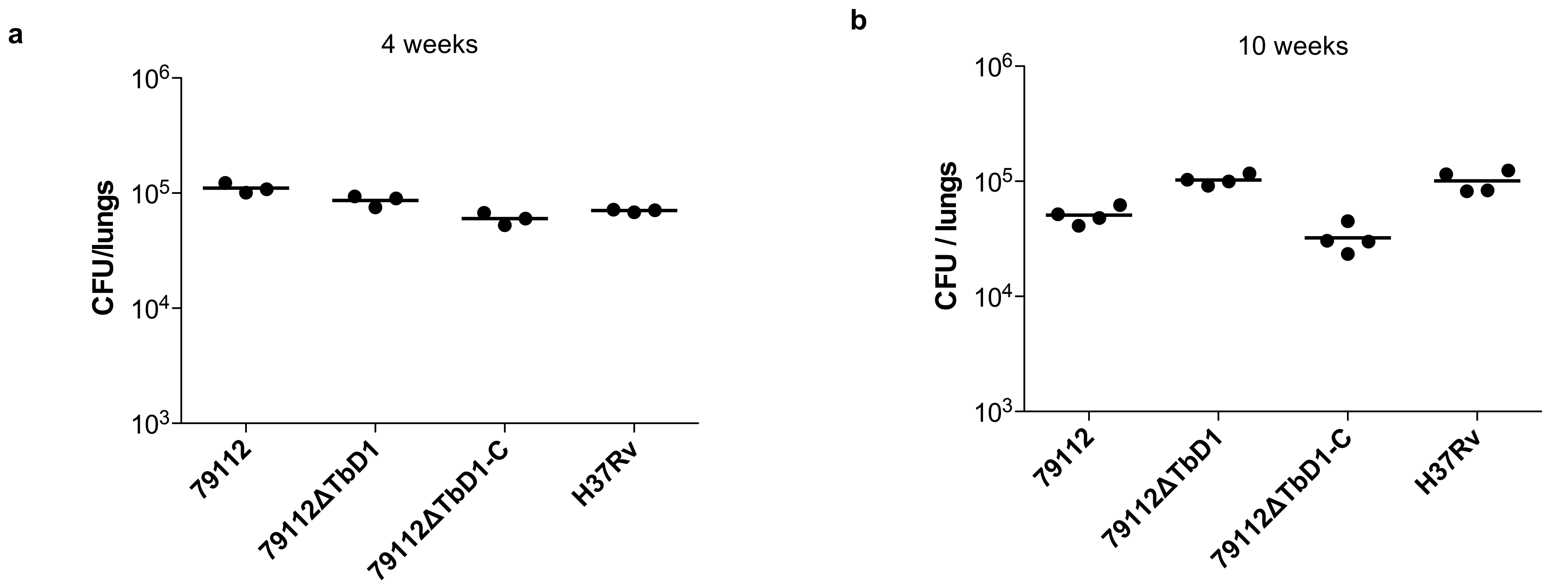

Supplementary Figure 8. In vivo growth characteristics of *M. tuberculosis* 79112 and isogenic TbD1-deleted and complemented strains in the lungs of C3HeB/FeJ mice. The bacterial load in the lungs of C3HeB/FeJ mice obtained at 4 (a) or 10 (b) weeks after aerosol low-dose infection (10 CFU/lungs) is shown. The figure depicts the single data points of CFU values recovered from lungs of infected mice in a representative experiment performed with 3 (4 weeks) or 4 (10 weeks) mice/group.

Supplementary Figure 9a. Histological preparations of lungs from C3HeB/FeJ mice, at 14 weeks post infection with TbD1-intact and TbD1-deleted *M. tuberculosis* strains. After fixation in 10% neutral buffered formalin for 24h-48h, organs were embedded in paraffin for 4-µm sectioning according to standard procedures (1) and stained in in hematoxylin and eosin (HE). Stained slides were evaluated with axio scan.Z1 and Zen software (Zeiss).

(1) Crosby K, Simendinger J, Grange C, Technol MF-CS, 2016 undefined. Immunohistochemistry protocol for paraffin-embedded tissue section-advertisement. jove.com [Internet]. [cited 2019 Mar 27]; Available from: https://www.jove.com/video/5064/immunohistochemistry-protocol-for-paraffin-embedded-tissue-sections

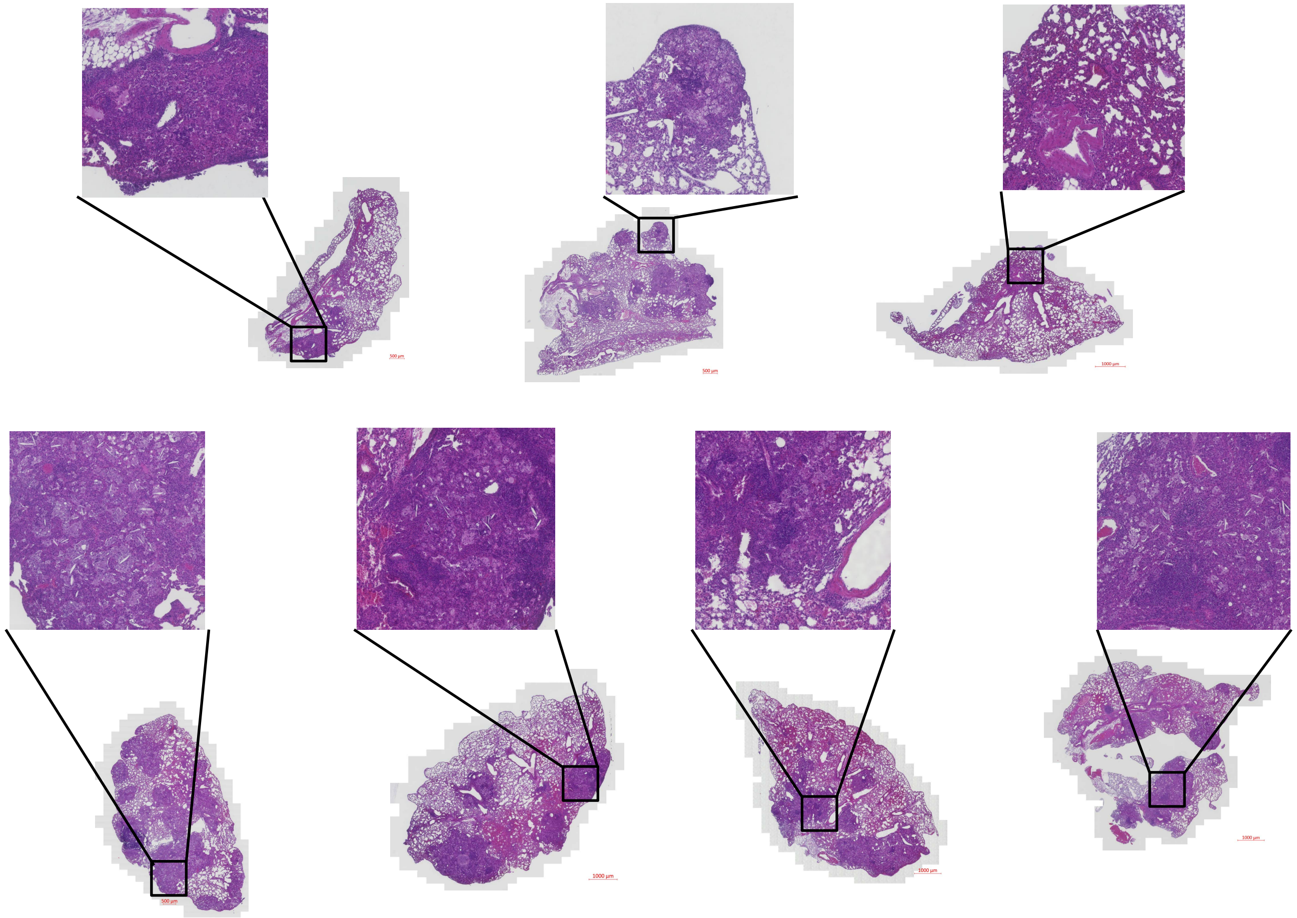

79112

79112ΔTbD1

Original magnification x2

Supplementary Figure 9b. Histological preparations of lungs from C3HeB/FeJ mice, at 14 weeks post infection with TbD1-intact and TbD1-deleted *M. tuberculosis* strains. After fixation in 10% neutral buffered formalin for 24h-48h, organs were embedded in paraffin for 4-µm sectioning according to standard procedures (1) and stained in in hematoxylin and eosin (HE). Stained slides were evaluated with axio scan.Z1 and Zen software (Zeiss).

(1) Crosby K, Simendinger J, Grange C, Technol MF-CS, 2016 undefined. Immunohistochemistry protocol for paraffin-embedded tissue section-advertisement. jove.com [Internet]. [cited 2019 Mar 27]; Available from: https://www.jove.com/video/5064/immunohistochemistry-protocol-for-paraffin-embedded-tissue-sections

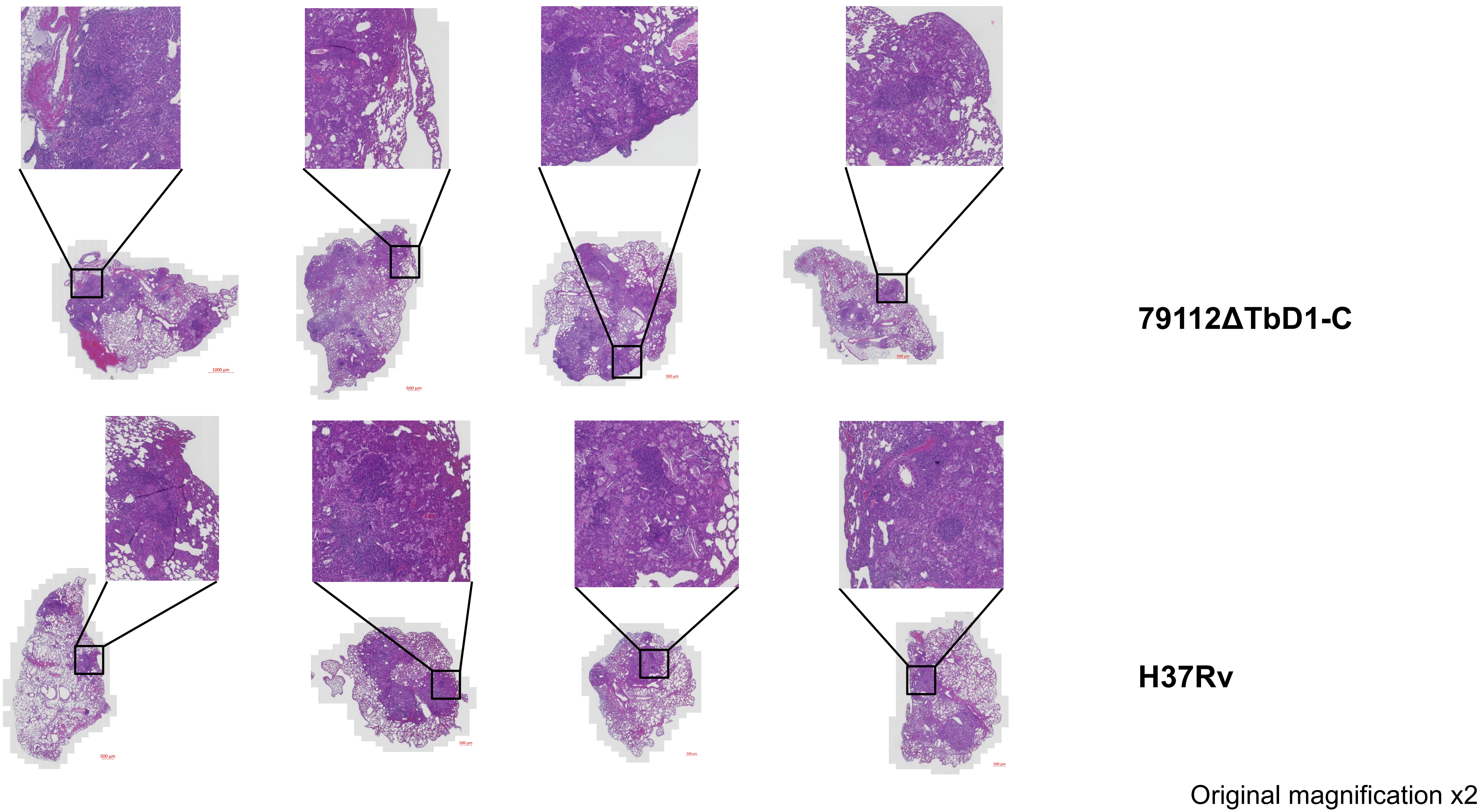

Supplementary Figure 10. Thin layer chromatography of extractible lipids in indicated solvent mixtures. Lipid preparations were obtained from four different WT and mutant *M. tuberculosis* strains grown under aerobic or hypoxic conditions for 3 weeks. TDM= trehalose dimycolate; TMM= trehalose monomycolate; CL= cardiolipin;
PE= phosphatidylethanolamine; PI= phosphatidylinositol; PIM= phosphatidylinositol mannoside;
AC_1PIM_2= Acyl phosphatidylinositol mannoside di-mannose ; AC_2PIM_2 Di-acyl phosphatidylinositol mannoside di-mannose

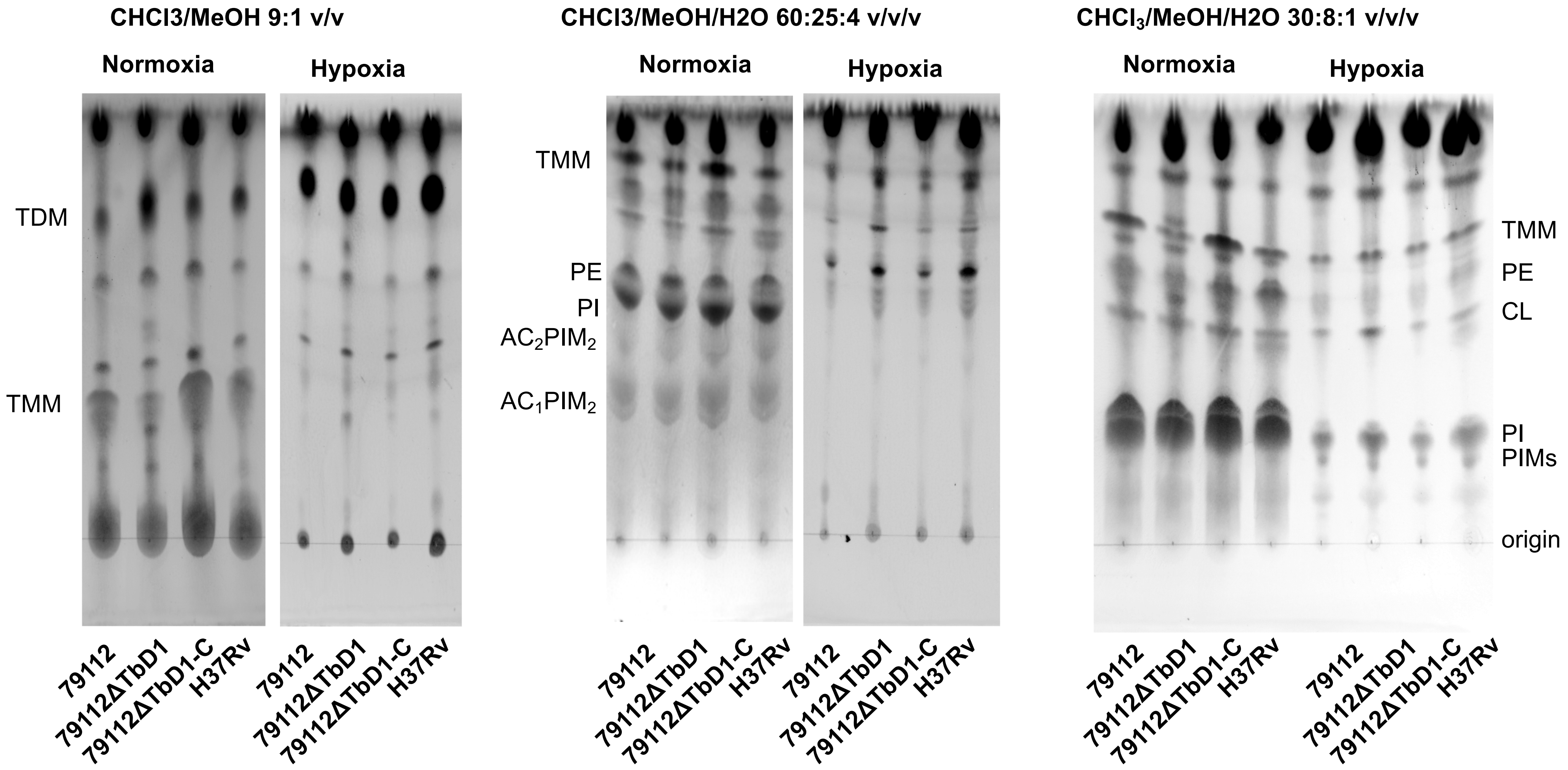

Supplementary Figure 11. Negative ion mode MALDI-ToF mass spectra of five *M. tuberculosis* strains under normoxia and hypoxia. PI= phosphatidylinositol; PIM= phosphatidylinositol mannoside; AC_1PIM_2= Acyl phosphatidylinositol mannoside di-mannose ; AC_2PIM_2 Di-acyl phosphatidylinositol mannoside di-mannose; AC_1PIM_6=Triacylated phosphatidyl-myo-inositol hexamannoside;

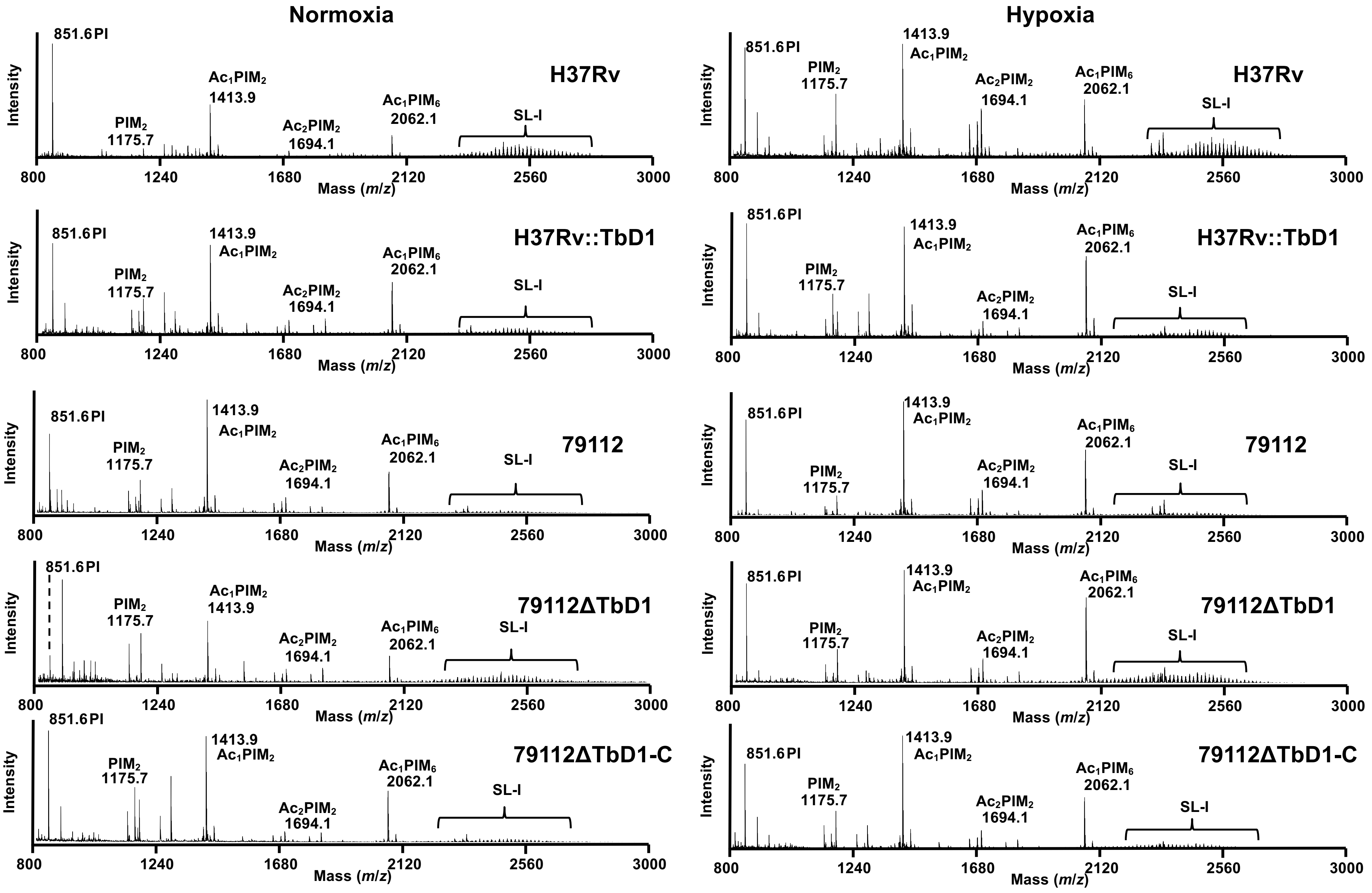

Supplementary Figure 12. MALDI-ToF mass spectra of mycolic acid methyl esters (MAMEs) obtained from four *M. tuberculosis* strains grown under normoxia and hypoxia.

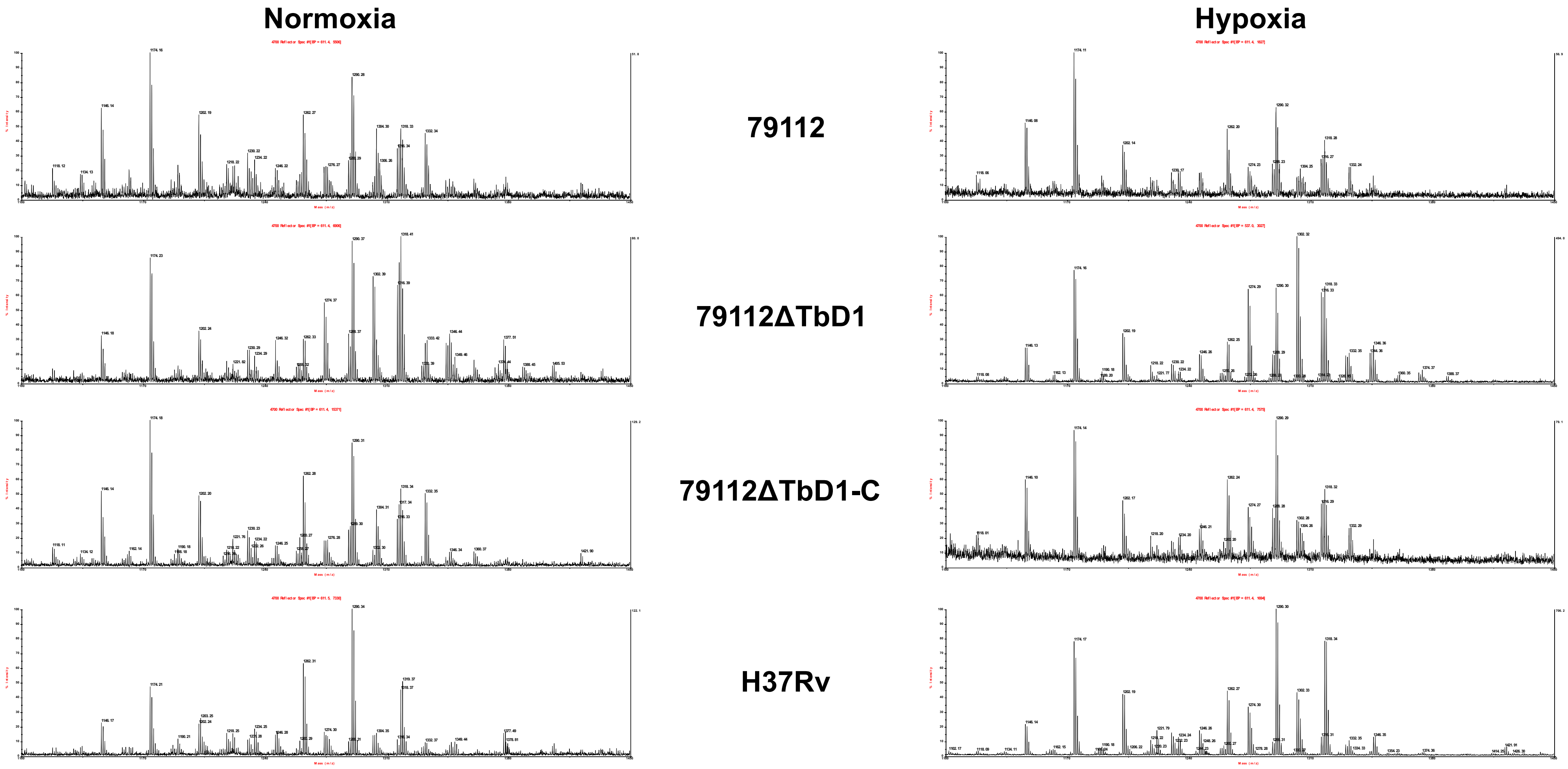

Supplementary Figure 13. Sensitivity of TbD1-intact and TbD1-deleted *M. tuberculosis* strains to different ROI and RNI.

a and **b**. Susceptibility of Tb36 and 79112 ancestral TbD1-intact strains to cumene hydroperoxide (a) and plumbagine (b) in comparison with the modern TbD1-deleted H37Rv reference strain. For each strain, the sensitivity to both compounds was evaluated by determination of the diameter of the growth inhibition zone (cm) in disk diffusion assays. Panels display single data, mean and standard deviation of diameter values measured in three independent samples for each strain. *: P< 0.05; ns: not significant, one-way Anova with Bonferroni post hoc test.

c. Survival of Tb36, 79112 and H37Rv strains in the presence of different RNI: 10 mM SNP and 1 mM or 10 mM $NaNO_2$. Bacterial cultures were incubated in Middlebrook 7H9 medium supplemented with 10 mM SNP (SNP survival assays), or in previously acidified Middlebrook 7H9 medium (pH 5.5) alone or supplemented with 1 mM or 10 mM $NaNO_2$ ($NaNO_2$ survival assays). After 1 or 4 days of incubation, the CFU number was determined. The susceptibility of each strain at different time points was expressed as percentage of survival relative to the time 0. (*: P< 0.05; ns: not significant,). For each strain, single data, mean and standard deviation of survival percentages obtained in three independent control or stress-exposed cultures are showed. The differences between TbD1-intact and TbD1-deleted *M. tuberculosis* strains versus *M. tuberculosis* H37Rv were not statistically significant (one-way Anova with Bonferroni post hoc test).

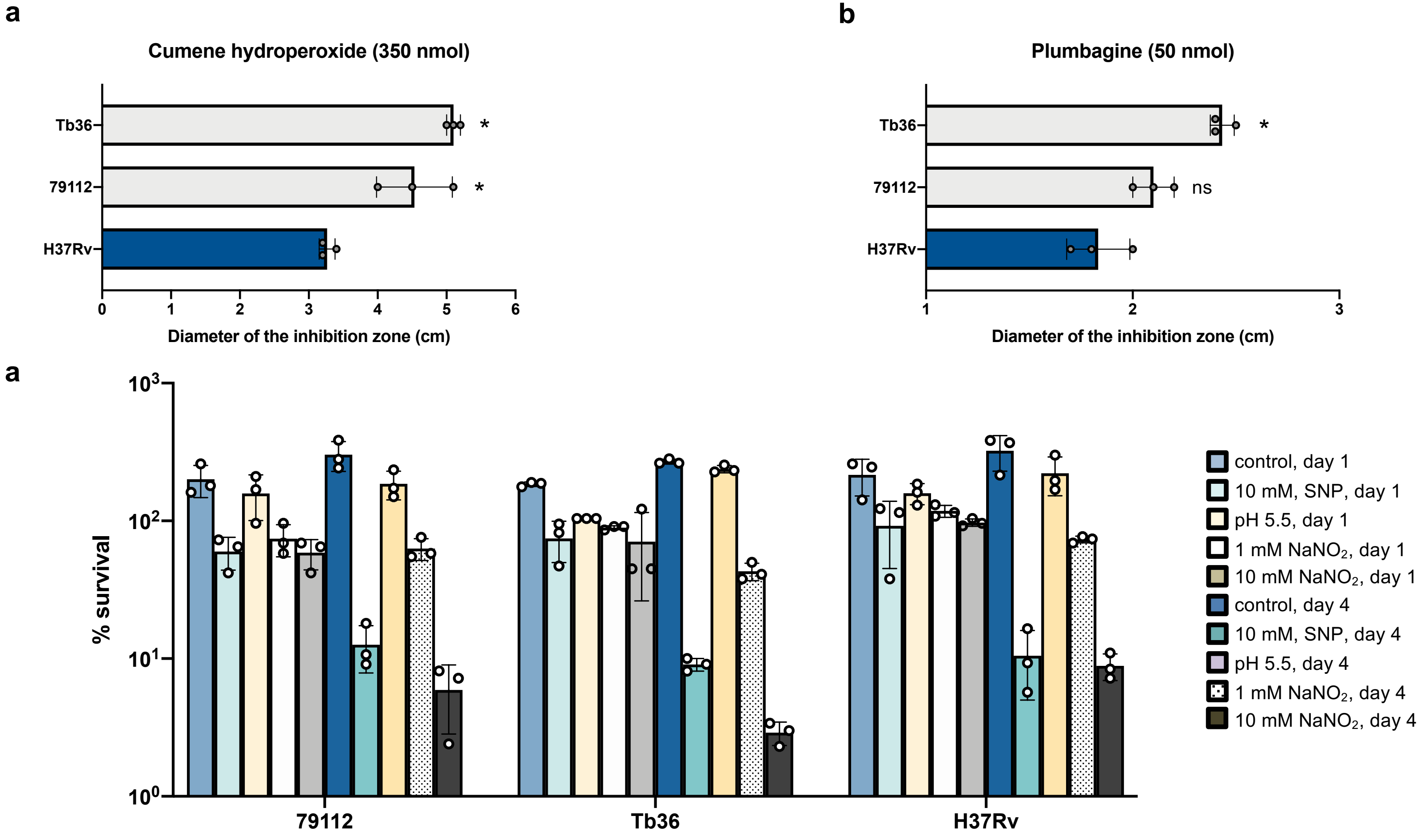

Supplementary Figure 14. Expression levels of *katG*, *ahpC*, *ahpD*, *sigJ* genes in TbD1 intact *M. tuberculosis* Tb36 and 79112 strains and the H37Rv control strain. Gene expression was evaluated both in basal conditions (untreated culture) and after exposure (20 min) to 10 mM H_2O_2. For each gene, the expression level in each strain is reported as the ratio between the expression level in *M. tuberculosis* H37Rv untreated, used as reference. For each strain, values were normalized to the level of *sigA*. The figure reports the data obtained in a representative experiment, where gene expression in different samples was tested in triplicate (or in duplicate, for the *sigJ* expression analysis in H_2O_2 exposed cultures).

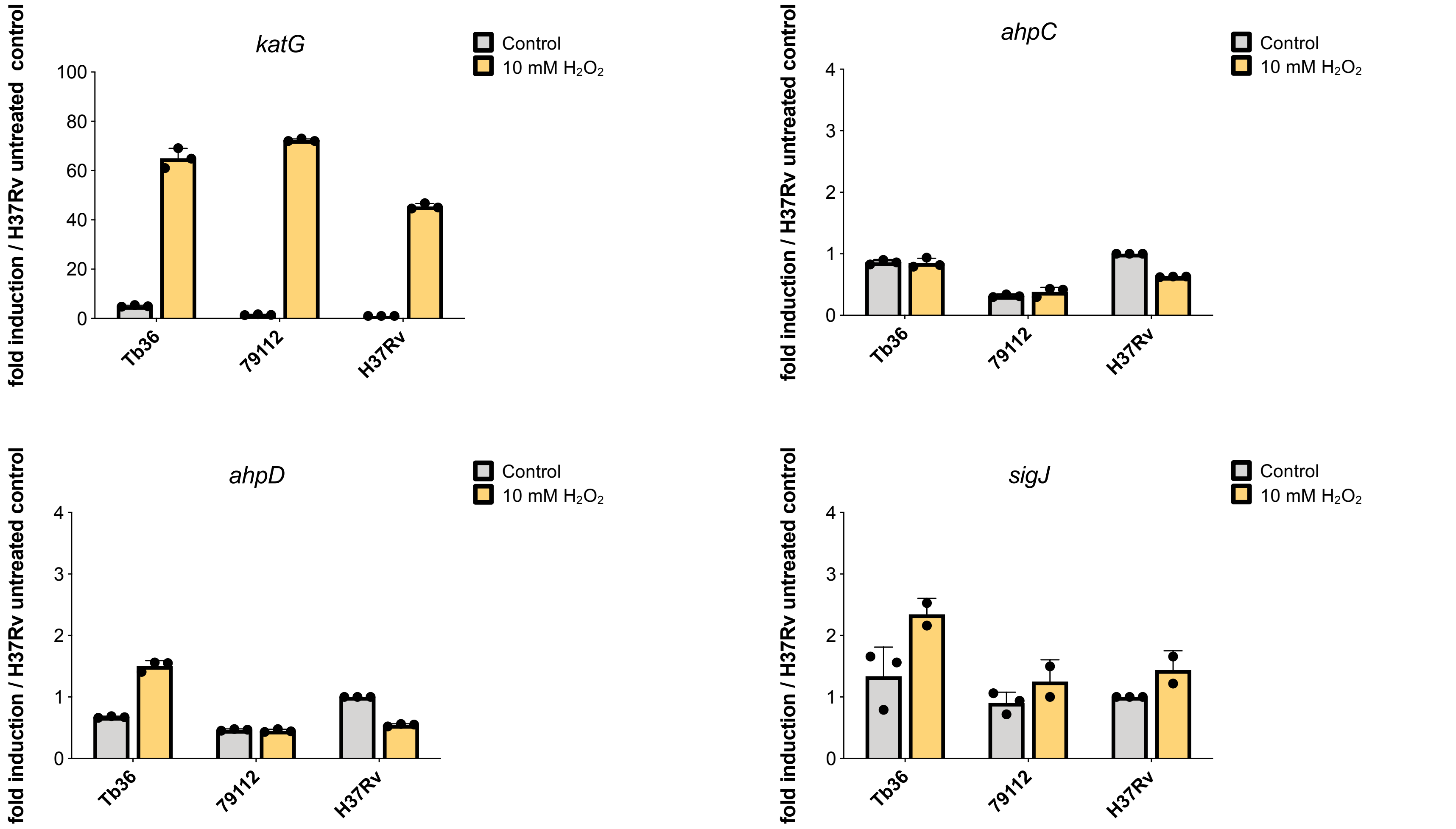

Supplementary Figure 15. Expression levels of *dosR*, *dosS*, *dosT*, genes in TbD1 intact Tb36 and 79112 strains and the H37Rv control strain in the Wayne model of progressive oxygen depletion. For each strain, gene expression was evaluated at different time points of growth/survival (5, 10 and 15 days). For each gene, the expression level is reported as the ratio between the expression level in *M. tuberculosis* H37Rv, used as reference. For each strain, values were normalized to the level of *sigA*.
The figure shows the data obtained in a representative experiment, where gene expression in each culture was tested in triplicate.

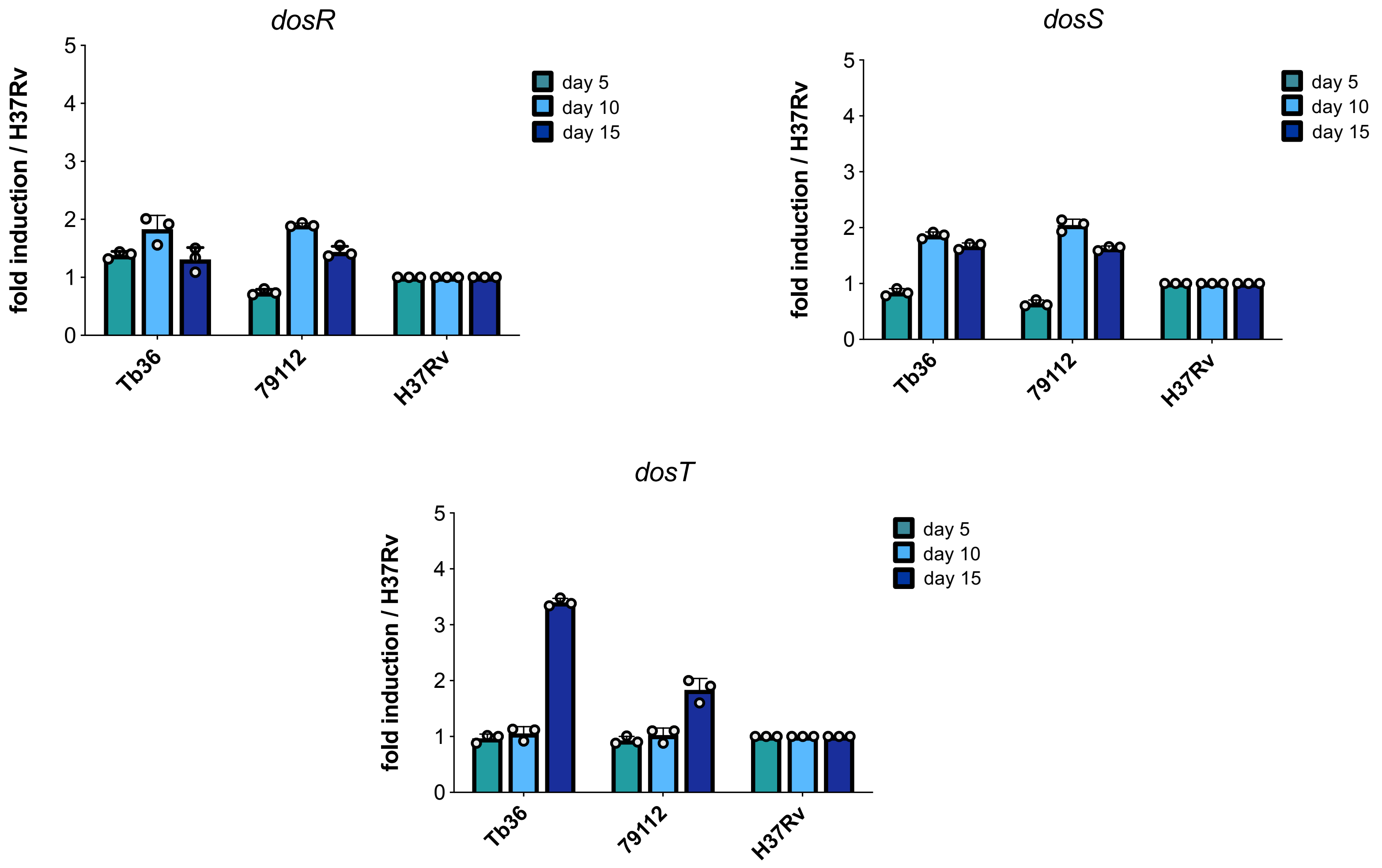

Supplementary Information - Raw data (uncropped gels):

Fig. 1

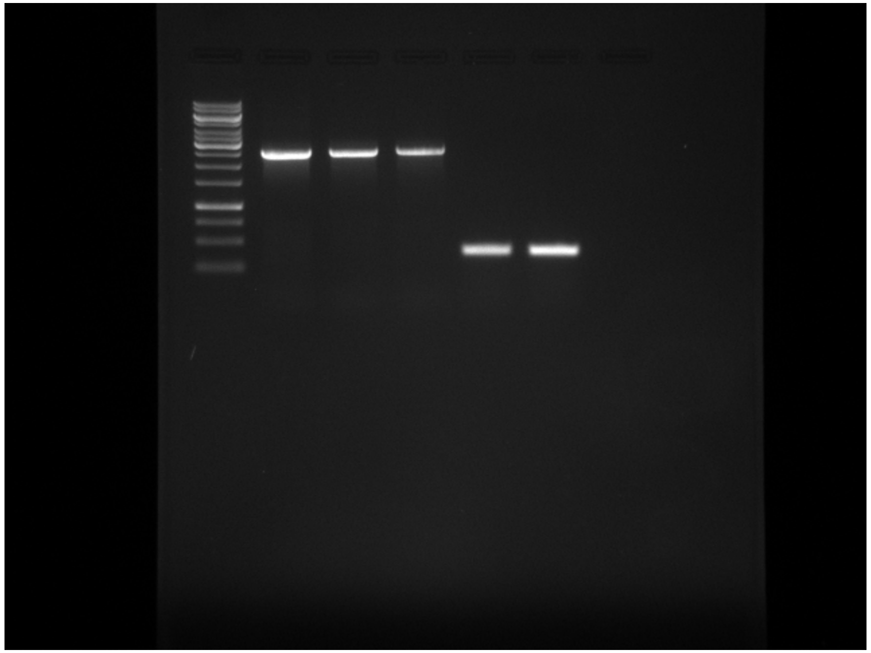

Fig. 3A

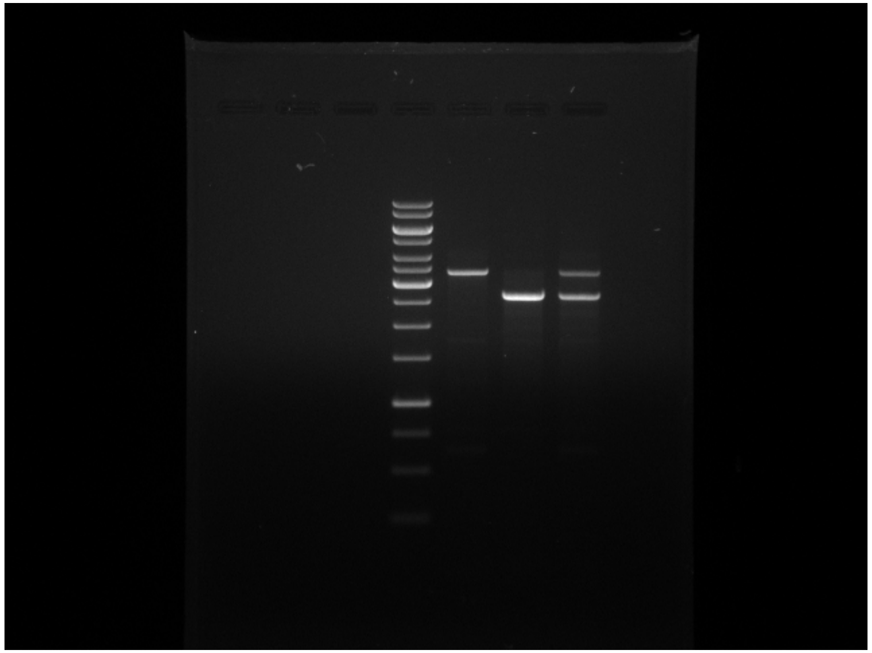

Fig. 3B

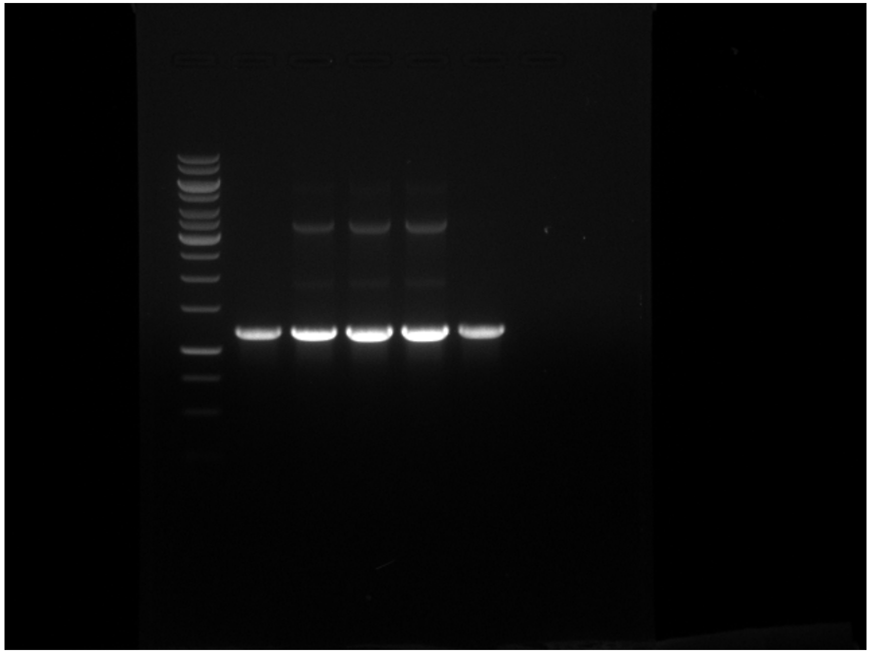

Fig. 4B

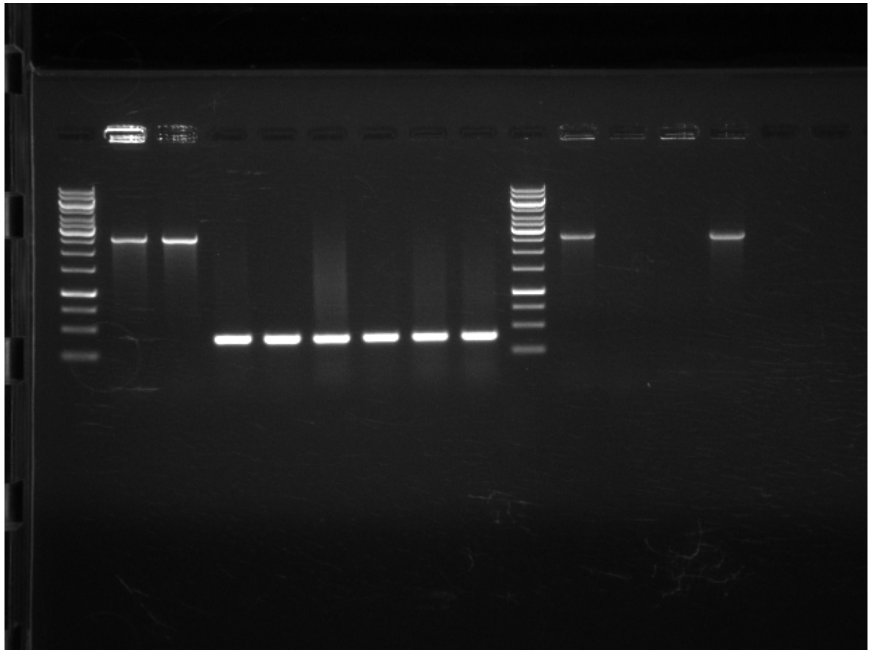

Supplementary Table 1. Sequences of primers used in this study

Name	Nucleotide sequence 5'-3'	Use
TbD1-PART1-F<br>TbD1-PART4-R	**GATCCACCGGTTGAGTCAGC**<br>**GCATAGATCCCGGACATGGT**	Screening of *M.tuberculosis* strains for detection of the intact or deleted TbD1 locus
Spe-TbD1-up-F<br>Xba-TbD1-up-R	**GGACTAGTTCTGTCGGGAGGTGGGCCAA**<br>**GCTCTAGAGCACCATCCAGCCGCGTTTG**	Amplification of the 5'-TbD1 homologous region for construction of the 79112ΔTbD1 mutant strain
Xba-TbD1-d-F<br>Not-TbD1-d-R	**GCTCTAGAGATCCGTCTTTGACACGATCGA**<br>**ATAAGAATGCGGCCGCTGTCCTTGAAGGTGGCGGCC**	Amplification of the 3'-TbD1 homologous region for construction of the 79112ΔTbD1 mutant strain
Xba-kana-F<br>Xba-kana-R	**GCTCTAGAGCTGCAAGGCGATTAAGT**<br>**GCTCTAGAATTAGGCACCCCAGGCTTTA**	Amplification of the kanamycin resistance gene from the pUC4k plasmid
sc-TbD1-F<br>sc-TbD1-R	**TTCATCGAGGGCATCCGCCA**<br>**ATGATCATCATGATGAGCAAAATCA**	Genotypic characterization of 79112ΔTbD1 and its complemented derivative strain
mmpS6 F<br>mmpL6 R	**GGACTAGTTCTGTCGGGAGGTGGGCCAA**<br>**GCTCTAGAGCACCATCCAGCCGCGTTTG**	Genotypic characterization of H37Rv::TbD1 complemented strains
R16S-F<br>R16S-R	**AGAGTTTGATCCTGGCTCAG**<br>**CCGTCAATTCCTTTGAGTTT**	Control of RNA preparations for DNA contamination
qt-sigA F<br>qt-SigA-R	**AGTCGGAGGCCCTGCGTCAA**<br>**GCCAGCCTCGATCCGCTTGG**	Quantitative-RT-PCR
qt-katG-F<br>qt-katG-R	**CCGGTAAGGACGCGATCACC**<br>**CCAGCAGGGCTCTTCGTCAG**	Quantitative-RT-PCR
qt-ahpC-F<br>qt-ahpC-R	**AAGCCGCAGGTGTCCTCAAC**<br>**CTCATCGACGTTGCGTCCCA**	Quantitative-RT-PCR
qt-ahpD-F<br>qt-ahpD-R	**GAGCTCAATCACCCGCAGCA**<br>**GCTTCAGCGCCAATGTCAGC**	Quantitative-RT-PCR
qt-sigJ-F<br>qt-sigJ-R	**GAATCAGCTGGCGCTGGTCA**<br>**GCCGGTGAACTTGTCGGGAT**	Quantitative-RT-PCR
qt-dosR-F<br>qt-dosR-R	**ACTGTGCCGCGATCTGT**<br>**TCCCTTGATGTCTTTGACGA**	Quantitative-RT-PCR
qt-dosS-F<br>qt-dosS-R	**AATGCGTCCACTGCGTCACA**<br>**ACGATAGCGCGTAGGGTTGC**	Quantitative-RT-PCR
qt-dosT-F<br>qt-dosT-R	**CTCATCGCCGAGGAAGCGTT**<br>**TCTCTCCGGCCACCTCTACG**	Quantitative-RT-PCR
