# Supplementary material for: TbD1 deletion as a driver of the evolutionary success of modern epidemic Mycobacterium tuberculosis lineages
Source: Nat Commun. 2020 Feb 4;11:684. doi: 10.1038/s41467-020-14508-5 (PMC7000671; doi:10.1038/s41467-020-14508-5)
Supplement: Supplementary file 3 — Reporting Summary [file 41467_2020_14508_MOESM3_ESM.pdf]

nature research

Corresponding author(s): Daria Bottai
Roland Brosch

Last updated by author(s): 22-12-2019

# Reporting Summary

Nature Research wishes to improve the reproducibility of the work that we publish. This form provides structure for consistency and transparency in reporting. For further information on Nature Research policies, see Authors & Referees and the Editorial Policy Checklist.

## Statistics

For all statistical analyses, confirm that the following items are present in the figure legend, table legend, main text, or Methods section.

| n/a | Confirmed | |
|---|---|---|
| ☐ | ☒ | The exact sample size (*n*) for each experimental group/condition, given as a discrete number and unit of measurement |
| ☐ | ☒ | A statement on whether measurements were taken from distinct samples or whether the same sample was measured repeatedly |
| ☐ | ☒ | The statistical test(s) used AND whether they are one- or two-sided<br>*Only common tests should be described solely by name; describe more complex techniques in the Methods section.* |
| ☐ | ☒ | A description of all covariates tested |
| ☐ | ☒ | A description of any assumptions or corrections, such as tests of normality and adjustment for multiple comparisons |
| ☐ | ☒ | A full description of the statistical parameters including central tendency (e.g. means) or other basic estimates (e.g. regression coefficient) AND variation (e.g. standard deviation) or associated estimates of uncertainty (e.g. confidence intervals) |
| ☐ | ☒ | For null hypothesis testing, the test statistic (e.g. *F*, *t*, *r*) with confidence intervals, effect sizes, degrees of freedom and *P* value noted<br>*Give P values as exact values whenever suitable.* |
| ☒ | ☐ | For Bayesian analysis, information on the choice of priors and Markov chain Monte Carlo settings |
| ☒ | ☐ | For hierarchical and complex designs, identification of the appropriate level for tests and full reporting of outcomes |
| ☒ | ☐ | Estimates of effect sizes (e.g. Cohen's *d*, Pearson's *r*), indicating how they were calculated |

*Our web collection on statistics for biologists contains articles on many of the points above.*

## Software and code

Policy information about availability of computer code

| | |
|---|---|
| Data collection | N/A |
| Data analysis | Data analysis was performed using GraphPad Prism program, versions 5 and 8.3.0, and the IBM SPSS web report Application server |

For manuscripts utilizing custom algorithms or software that are central to the research but not yet described in published literature, software must be made available to editors/reviewers. We strongly encourage code deposition in a community repository (e.g. GitHub). See the Nature Research guidelines for submitting code & software for further information.

## Data

Policy information about availability of data

All manuscripts must include a data availability statement. This statement should provide the following information, where applicable:

- Accession codes, unique identifiers, or web links for publicly available datasets
- A list of figures that have associated raw data
- A description of any restrictions on data availability

Genome assemblies of Mtb 79112, 79499 and Tb36 have been deposited in the EMBL/ENA database under the study accession number PRJEB30653. https://www.ebi.ac.uk/ena/browser/view/PRJEB30653

# Field-specific reporting

Please select the one below that is the best fit for your research. If you are not sure, read the appropriate sections before making your selection.

☒ Life sciences ☐ Behavioural & social sciences ☐ Ecological, evolutionary & environmental sciences

# Life sciences study design

All studies must disclose on these points even when the disclosure is negative.

| | |
|---|---|
| Sample size | Samples sizes for animal experiments were chosesn according to the needs of statistical analyses and the ethical principles of 3Rs |
| Data exclusions | No data have been excluded |
| Replication | Experiments were replicated  as stated in the methods section and figure legends |
| Randomization | There was no randomization, specially selected samples were included in the study as stated in the methods sections |
| Blinding | No blinding was performed. |

# Reporting for specific materials, systems and methods

We require information from authors about some types of materials, experimental systems and methods used in many studies. Here, indicate whether each material, system or method listed is relevant to your study. If you are not sure if a list item applies to your research, read the appropriate section before selecting a response.

## Materials & experimental systems

| n/a | Involved in the study | |
|---|---|---|
| ☒ | ☐ | Antibodies |
| ☐ | ☒ | Eukaryotic cell lines |
| ☒ | ☐ | Palaeontology |
| ☐ | ☒ | Animals and other organisms |
| ☒ | ☐ | Human research participants |
| ☒ | ☐ | Clinical data |

## Methods

| n/a | Involved in the study | |
|---|---|---|
| ☒ | ☐ | ChIP-seq |
| ☒ | ☐ | Flow cytometry |
| ☒ | ☐ | MRI-based neuroimaging |

## Eukaryotic cell lines

Policy information about cell lines

| | |
|---|---|
| Cell line source(s) | ATCC |
| Authentication | Standard cell lines were used, no particular authentification process was conducted |
| Mycoplasma contamination | Cells lines were not tested for Mycoplasma contamination |
| Commonly misidentified lines (See ICLAC register) | N/A |

## Animals and other organisms

Policy information about studies involving animals; ARRIVE guidelines recommended for reporting animal research

| | |
|---|---|
| Laboratory animals | Laboratory animals used in the study involved: Five/eight week-old outbred Hartley guinea-pigs (300g) (Charles River),  six/eight-week-old female C57BL/6 mice (Charles River), and  six/eight-week-old male/female C3HeB/FeJ mice (originally purchased from Jackson Laboratories). |
| Wild animals | The study did not involve wild animals |
| Field-collected samples | The study did not involve samples collected from the field |
| Ethics oversight | All animal studies were performed in agreement with European and French guidelines (Directive 86/609/CEE and Decree 87–848 of 19 October 1987). The study received the approval by the Institut Pasteur Safety Committee (Protocol 11.245) and the ethical approval by local ethical committees “Comité National de Réflexion Ethique sur l'Expérimentation Animale N° 59 (CNREEA)” or “Comité d’Ethique en Experimentation Animale Institut Pasteur N° 89 (CETEA)” (CNREEA 2012-0061; CETEA 2013-0036, CETEA dab160018, CETEA dab180023). |

Note that full information on the approval of the study protocol must also be provided in the manuscript.
